# Effect of Lentivirus-Mediated miR-499a-3p on Human Umbilical Vein Endothelial Cells

**DOI:** 10.1155/2020/9372961

**Published:** 2020-08-26

**Authors:** Huilei Zheng, Juan Li, Ying Chen, Danping Gong, Jianlin Wen, Lanxian Mai, Zhiyu Zeng

**Affiliations:** ^1^Department of Medical Examination & Health Management, First Affiliated Hospital of Guangxi Medical University, Nanning, Guangxi 530021, China; ^2^Guangxi Key Laboratory of Precision Medicine in Cardio-cerebrovascular Diseases Control and Prevention & Guangxi Clinical Research Center for Cardio-cerebrovascular Diseases, Nanning, Guangxi, China; ^3^Department of Population Health Science, Duke University School of Medicine, Durham, North Carolina, USA; ^4^Department of Electrocardiography, Affiliated Hospital of Youjiang Nationalities Medical College, Baise, China; ^5^Intensive Care Unit, Guangxi Medical University Affiliated Tumor Hospital & Oncology Medical College & Guangxi Cancer Institute & Tumor Hospital & Cancer Center, Nanning, China; ^6^Elderly Cardiology Ward, First Affiliated Hospital of Guangxi Medical University, Nanning, Guangxi, China; ^7^Cardiac Surgery Intensive Care Unit, First Affiliated Hospital of Guangxi Medical University, Nanning, Guangxi, China; ^8^Disciplinary Construction Office, First Affiliated Hospital of Guangxi Medical University, Nanning, Guangxi, China

## Abstract

**Objective:**

To explore the possible role of miR-499a-3p in the function of primary human umbilical vein endothelial cells (HUVECs) and the expression of ADAM10 in primary HUVEC.

**Method:**

miR-499a-3p was first transfected into primary HUVECs via lentivirus vector. The viability, proliferation, and migration of stable transfected primary HUVEC were then determined by flow cytometry, CCK8 assays, scratch tests, and Transwell tests. The transcription of miR-499a-3p and ADAM10 was examined by reverse transcription-polymerase chain reaction (RT-PCR), and the expression of ADAM10 was examined by Western blot (WB).

**Results:**

After transfection, miR-499a-3p transcription was significantly increased (*P* < 0.01), compared to the blank and nonspecific control (NC) groups, while both ADAM10 transcription and expression were significantly decreased (*P* < 0.05). In contrast, in the inhibitors group, miR-499a-3p transcription was significantly reduced (*P* < 0.05) whereas both ADAM10 transcription and expression were significantly increased (*P* < 0.05). The viability, proliferation, and migration of primary HUVECs were significantly impaired (*P* < 0.05) by the transfection of miR-499a-3p but enhanced by miR-499a-3p inhibitors (*P* < 0.05).

**Conclusions:**

Upregulation of miR-499a-3p transcription will inhibit the expression of ADAM10 in HUVECs; cell migration and proliferation, however, promote apoptosis. And reverse effects were established by downregulation of miR-499a-3p transcription. All these effects may be achieved by regulating the transcription and expression of ADAM10. These results combined suggested that miR-499a-3p may affect the proliferation, migration, and apoptosis of endothelial cells and regulate AS by regulating ADAM10. miR-499a-3p may become a candidate biomarker for the diagnosis of unstable angina pectoris (UA).

## 1. Introduction

Coronary artery disease (CAD) refers to coronary atherosclerosis that narrows or blocks the lumen of blood vessels and/or is caused by damage or necrosis upon myocardial ischemia and hypoxia due to functional changes in coronary arteries (spasms) of heart disease, leading to asymptomatic myocardial ischemia, angina, myocardial infarction, ischemic heart disease, and sudden death [1–3]. CAD is the third leading cause of death in the world, causing 17.8 million deaths every year [4]. Unstable angina pectoris (UA), a severe type of CAD, is a clinical syndrome between chronic stable angina and acute myocardial infarction (AMI) [5, 6]. The changes in patients' conditions are unstable, difficult to predict, and high risk [7]. The release of the traditional laboratory indicator cardiac troponin (CTN) was delayed in the development of UA, while the highly sensitive cardiac troponin was equally sensitive to noncoronary pain [8], leading to a high false-positive rate and poor specificity in the diagnosis of UA [9] as it could not accurately distinguish myocardial infarction from UA [8]. Due to the lack of rapid, specific, and effective detection indicators, the incidence of AMI in UA patients within one year can reach 12%-13%, and the mortality rate is as high as 3%-18%. UA has become one of the biggest problems that seriously threaten public health. Therefore, it is urgent to find novel molecular markers with high specificity and sensitivity in the diagnosis and prognosis of UA.

The basic pathological mechanism of CAD is atherosclerosis (AS). The abnormal proliferation and migration of vascular endothelial cells (VECs) and smooth muscle cells (VSMCs) play a central role in the development of AS. Current medical studies have shown that the pathogenesis of UA is mainly related to vascular endothelial injury, platelet activation, inflammation, and other factors causing coronary atherosclerotic plaque rupture and secondary thrombosis [10], which are all closely related to endothelial cell dysfunction [11].

MicroRNAs (miRNAs) are a class of single-stranded, noncoding small molecular RNAs that are highly evolutionarily conserved and negatively regulate the expression of target genes after transcription [12, 13]. miRNAs play an important role in the occurrence and development of CAD [14]. Recent studies have shown that some circulating miRNAs can stably exist in the peripheral circulatory system [12, 15–17], and some scholars have found that miRNAs can be used as potential biomarkers for UA [18–20].

Previous studies found that miR-499a-3p was highly expressed in UA patient serum compared with noncoronary heart disease patients with suspected angina pectoris, and the negative regulatory relationship between miR-499a-3p and ADAM10 was verified by luciferase reporter experiments. Xu et al. [21] found that miR-499a-3p can promote the proliferation and migration of endothelial cells and smooth muscle cells in AS by directly targeting myocardial cell enhancement factor 2C (MEF2C). ADAM10 is highly expressed in coronary arteries, myocardium, and atheromatous plaques and has been previously found to be related to AS promoting the proliferation, migration, and chemotaxis of endothelial cells and increasing vascular permeability and leukocyte penetration into the endothelium [22]. ADAM10 can also regulate the apoptosis of smooth muscle cells and macrophages, and the occurrence of apoptosis is closely related to plaque instability [23].

At present, there are few reports on the role of miR-499a-3p in UA and its relationship with ADAM10. This study speculated that miR-499a-3p might affect endothelial cell proliferation, migration, and apoptosis by influencing ADAM10. Therefore, this study is aimed at constructing stably transfected cell lines with unregulated or downregulated expression of miR-499a-3p and at detecting the expression of miR-499a-3p and ADAM10 in the stably transfected cells and its effect on HUVEC function. This work provides clues for the mechanism of miRNAs affecting AS by regulating endothelial cells, provides a basis for finding markers for the early diagnosis of UA, and identifies a potential intervention target to avoid the occurrence of malignant coronary events.

## 2. Material and Methods

### 2.1. Cell Lines and Culture

Primary HUVECs were purchased from Zhongqiao Xinzhou Biological Co., Ltd. (Shanghai, China). HUVECs were maintained in F12K (Gibco, USA) with 10% FBS (Gibco, USA), Endothelial Cell Growth Supplement (ScienCell, USA), and 1% penicillin-streptomycin-amphotericin solution (Punosai Life Technology Co., Ltd., China).

293T cells were purchased from the Institute of Cell Sciences, Chinese Academy of Sciences (Shanghai, China) and grown in DMEM (Gibco, USA) supplemented with 10% FBS and 1% penicillin-streptomycin-amphotericin solution (Punosai Life Technology Co., Ltd., China).

### 2.2. Lentivirus Production

The lentivirus used was purchased from Shanghai Jikai Gene Chemical Technology Co., Ltd. The sequence of miR-499a-3p sequence (MIMAT0004772: AACAUCACAGCAAGUCUGUGCU) was synthesized according to the record in miRBase database. Restriction sites were introduced based on the characteristics of puromycin-resistant vectors GV280 and GV263 (Shanghai Jikai Gene, China), which also contains green fluorescent protein (GFP). The reverse complementary sequence was designed according to the above sequence. Then, *oligo*-primers with corresponding cleavage sites were also designed based on the precursor sequence encoding human miR-499a-3p in the database.

*Generation of linearized digested vectors*: the vectors GV280 and GV263 were digested in a 50 *μ*L reaction. Then, 41 *μ*L of double-distilled water and 25 *μ*L of 10x CutSmart Buffer were added to the system to purify 2 *μ*L of plasmid DNA. The reaction was mixed well. After a brief centrifugation, the mixture was incubated at 37°C for 180 min. The digested product was purified by agarose gel electrophoresis, and the target band was recovered quickly.

*Recombinant lentivirus vector and identification*: the designed pair of oligonucleotides was dissolved in annealing buffer, heated at 90°C in a water bath for 15 min, and cooled to room temperature to anneal the F chain with the R chain into double-stranded DNA. Using T4 DNA ligase (Shanghai Jikai Gene, China), the annealed oligonucleotides and double-digested linearized vector were ligated at 16°C for 2 hours. 10 *μ*L ligated product was added to 100 *μ*L DH5 competent cells (Shanghai Jikai Gene, China). After mixing, the cells were placed in an ice bath for 2 min, mixed with 500 *μ*L medium, and shaken at 37°C for 1 hour. The bacterial solution was inoculated on a plate containing antibiotics and incubated at 37°C for 12 hours. The transformed colonies were subjected to PCR identification. The PCR identification, the F primer bound to the vector, the single-stranded R primer annealed downstream, and the PCR system was 20 *μ*L. After identification, the positive clones were sent for sequencing identification and comparison analysis in the database.

*Lentivirus vector production and titer identification*: 293T were seeded in a 10 cm Petri dish and cultured until the coverage percentage reached ~80%. The culture medium was renewed with serum-free DMEM : F12 medium, and cells were cultured in normal conditions overnight. Then, the 293T cells were cotransfected with the constructed recombinant plasmid via packaging plasmids pHelper1.0, pHelper2.0 (Shanghai Jikai Gene, China), and Lipofectamine 2000. After 48~72 h, the transfection efficiency was observed under a fluorescence microscope, and the supernatant was collected to determine the virus titer.

### 2.3. Optimal Transfection Dose

To choose an optimal transfection dose, six doses were tested (MOI: 1, 5, 10, 20, 50, and 100). For each transfection dose, four different conditions were applied as follows: (i) complete medium, (ii) complete medium+polybrene, (iii) enhanced infection solution, and (iv) enhanced infection solution+polybrene group. For each condition, untransfected cells were adopted as the control to see whether cell growth will be affected by the condition.

### 2.4. Lentivirus Transfection

Primary HUVECs were used instead of human coronary VECs. Four groups were set as follows: (i) normal cells without any transfection (blank control, blank), (ii) cells transfected with blank vectors (nonspecific control, NC), (iii) cells transfected with miR-499a-3p (mimics), and (iv) cells transfected with miR-499a-3p inhibitors (inhibitors). Cells were first seeded in 6-well plates (6 × 10^4^ cells/well) and cultured in normal condition for 24 h and then transfected at MOI of 10. 48 h later, the expression was observed under a fluorescent microscope. Cells successfully transfected were screened out with 2 *μ*g/mL puromycin (Selleck, USA). After 10 days, all normal cells in the blank group died.

### 2.5. RT-PCR

Cells from each group were cultured in 6-well plates. When the density reached 90%, RioPlus was added to extract the total RNA of the cells, and the concentration and purity were detected. Total RNA was reverse transcribed to obtain cDNA, and PCR was performed in a 10 *μ*L reaction system. The reaction conditions were 37°C for 1 hour and 85°C for 5 minutes. U6 was selected as an internal reference, and the expression of miR-499a-3p was calculated using the 2^-*ΔΔ*Ct^ method. GAPDH was selected as an internal control, and the expression of ADAM10 was detected in the same way as above.

### 2.6. Western Blot

Total proteins were extracted after three days of proliferation when they have covered ~90% area, according to the instruction of the Mem-PER™ Plus Membrane Protein Extraction Kit (Thermo, USA). The protein content was quantified by the BCA method. Proteins were separated by SDS-PAGE (separation gel: 8%) first and then transferred to PVDF membranes (Bio-Rad, CA, USA). After blocking with 10% nonfat milk, the membranes were incubated with the specified primary antibodies at 4°C overnight: anti-CAMK1 antibody (25900-1-AP) (Proteintech, USA) and anti-GAPDH polyclonal antibody (10494-1-AP) (Proteintech, USA). Then, after incubation with the corresponding secondary antibodies for 2 h, the protein bands were visualized using Clarity Western ECL Substrate (Bio-Rad, CA, USA).

### 2.7. Cell Scratch Test

Migration of transfected HUVECs was firstly determined by a wound healing assay. Six-well plates were used in this assay, and each well was seeded with 5 × 10^4^ cells. Cells were allowed to grow under normal conditions overnight, and a scratch was made manually the next day with a 200 *μ*L pipette tip. Then, culture medium containing 2% serum was added and the cells were imaged. Following imagination, these were carried out at an interval of 24 h. The change of the scratch area during 48 h was calculated by the following formula: the migration area (*t*) = the area (0 h) − the area (*t*).

### 2.8. Transwell Migration Assays

Cell groups were cultured to ~95% coverage percentage. Complete medium containing 2% serum was applied for additional 24 h. Then, the cells were digested, centrifuged, and resuspended. 100 *μ*L cell suspension was added to the upper chamber (Millipore, USA), and 500 *μ*L of complete medium was added to the lower chamber. The cells were cultured in an incubator (5% CO_2_, 37°C) for 24 h. The chamber was removed, fixed with 4% paraformaldehyde for 15 min, air-dried, filled with 0.1% crystal violet staining solution, and air-dried. The nonmigrated cells on the upper basement membrane were carefully cleared. After rinsing with PBS for 3 times, the intact basement membrane was carefully removed and placed on a glass slide. Five images of the field of view were taken for counting.

### 2.9. Cell Proliferation Assays

Cell proliferation ability was detected by the CCK8 assay (Sangon Biotech Co., Ltd., China). At the end of transfection, cells were plated in 96-well plates (5 × 10^3^ cells/well) and then cultured at 37°C. Next, CCK8 solution was added according to the manufacturer's instructions at 24, 48, 72, 96, and 120 h, respectively. The cells were then cultured for another 1.5 h in an incubator (37°C). The optical density value at 450 nm (OD_450_) was detected.

### 2.10. Flow Cytometry Detection of Apoptosis

Cell apoptosis was measured by flow cytometry using Annexin V-APC/7-AAD (Lianke Biotech Co., Ltd., China). At the end of transfection, cells were digested with trypsin (2 mg/mL) and washed with PBS. Cells were resuspended in 500 *μ*L of binding buffer and double-stained with 7-AAD and APC-conjugated Annexin V for 35 min at 4°C in the dark according to the manufacturer's instructions. The samples were detected by a BD FACSCanto Flow Cytometer (BD Biosciences, San Jose, CA, USA). The data were analyzed by BD FACSDiva software.

### 2.11. Statistical Analysis

All experiments were performed in triplicate. SPSS 22.0 statistical software was used to analyze the data. All data are expressed as the mean ± standard deviation (*x* ± *s*) (*n* = 3). Statistical significance was determined by Student's *t*-test or one-way ANOVA. Differences were regarded statistically significant at *P* < 0.05.

## 3. Results

### 3.1. Optimal Transfection Dose

**Figure 1:**
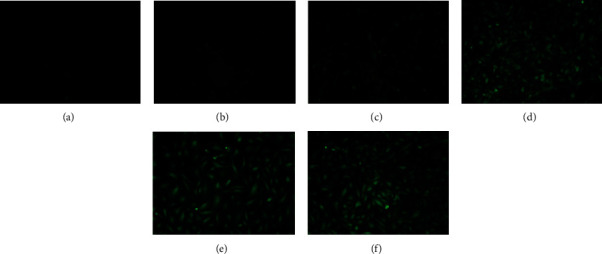
Fluorescence expression of blank controls at 72 h after transfection with different MOI. All cell groups were cultured in complete medium. (a) MOI = 1. (b) MOI = 5. (c) MOI = 10. (d) MOI = 20. (e) MOI = 50. (f) MOI = 100.

To choose an optimal dose, six transfection doses (MOI: 1, 5, 10, 20, 50, and 100) were examined. The fluorescence expressions of the blank controls after 72 h of transfection with different MOI are shown in Figure 1. The number of fluorescent puncta became obvious at a MOI of 10. Thus, the optimal transfection dose is set as complete medium + lentivirus titer at an MOI of 10.

### 3.2. Screening of Stably Transfected Cell Lines

All the cells in the control group treated with a concentration of puromycin at ≥2 ng/mL died while most of the cells treated with a concentration less than 2 ng/mL survived (data were not shown). The optimal concentration of puromycin was 2 ng/mL.

### 3.3. Transcription of miR-499a-3p and ADAM10

**Figure 2:**
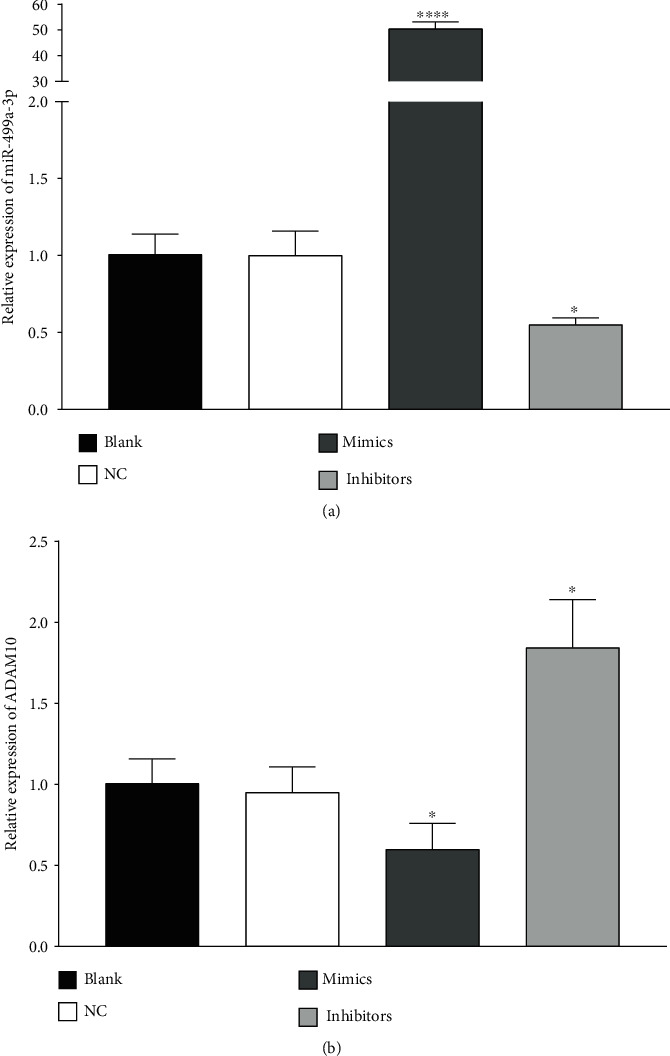
Transcription of miR-499a-3p and ADAM10 in different cell groups. (a) Transcriptions of miR-499a-3p after transfection in the blank, NC, mimics, and inhibitors groups were determined by RT-PCR. (b) Transcriptions of ADAM10 after transfection in the blank, NC, mimics, and inhibitors groups were determined by RT-PCR. All results were expressed as the mean ± standard deviation (*n* = 3).

**Figure 3:**
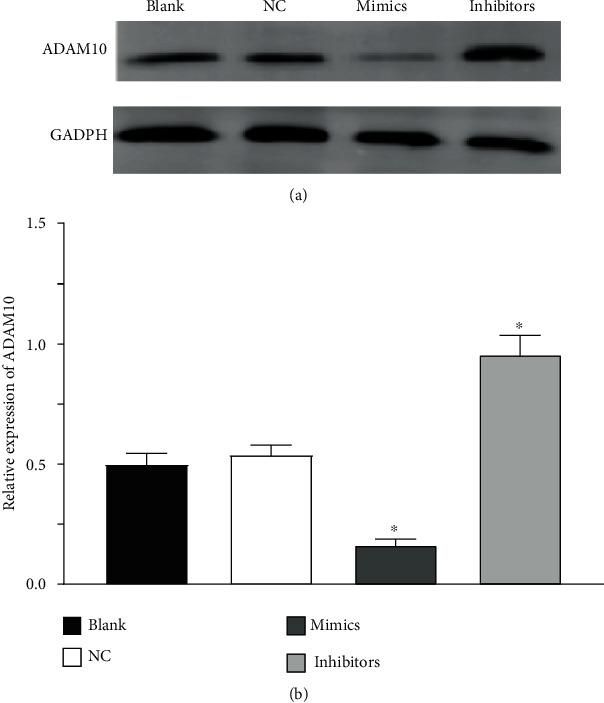
Expression of ADAM10 protein in different cell groups. (a) Western blot of ADAM10 expression in the blank, NC, mimics, and inhibitors groups after transfection of miR-499a-3p for 48 h. (b) Relative expression of ADAM10 in different groups. All results were expressed as the mean ± standard deviation (*n* = 3).

Transcriptions of miR-499a-3p and ADAM10 after transfection are shown in Figure 2, respectively. No significant difference was observed in the transcription levels of miR-499a-3p and ADAM10 in HUVECs between the blank and NC groups (*P* > 0.05), respectively. However, as shown in Figure 3, transcription of miR-499a-3p in the mimics group was significantly increased (*P* < 0.01), while that of ADAM10 was significantly decreased (*P* < 0.05) when compared with the NC group. In the inhibitors group, the transcription level of miR-499a-3p was significantly reduced (*P* < 0.05) and that of ADAM10 was significantly increased (*P* < 0.05).

### 3.4. Expression of ADAM10

There was no significant difference in the expression of ADAM10 between the blank and NC groups (*P* > 0.05). Compared with that of the NC group, the expression of ADAM10 decreased in the mimics group (*P* < 0.05), and the expression of ADAM10 in the inhibitors group increased (*P* < 0.05), as shown in Figure 3.

### 3.5. Cell Scratch Test Results

**Figure 4:**
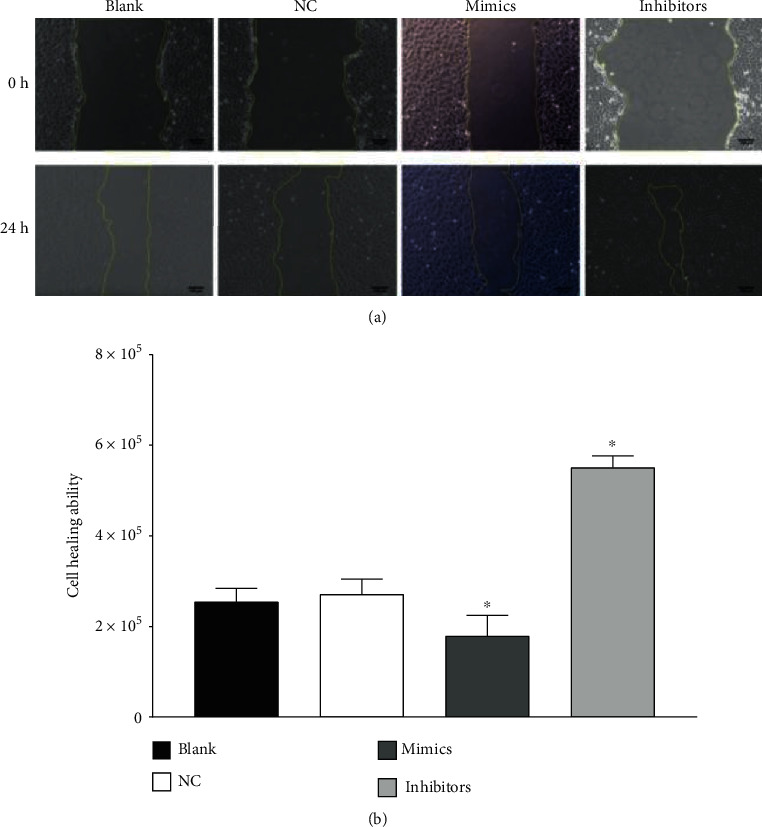
Effect of miR-499a-3p on the migration of HUVECs. (a) Schematic show (magnitude: 100x) of the effects of miR-499a-3p on the migration ability of HUVECs examined by scratch assays after transfection for 24 h. (b) The healing area of the blank, NC, mimics, and inhibitors group after transfection for 24 h. It is calculated by the difference value of the scratching area at *t* = 0 and *t* = 24 h. Three random sections from the scratching were chosen and compared for each cell group (*n* = 3).

**Figure 5:**
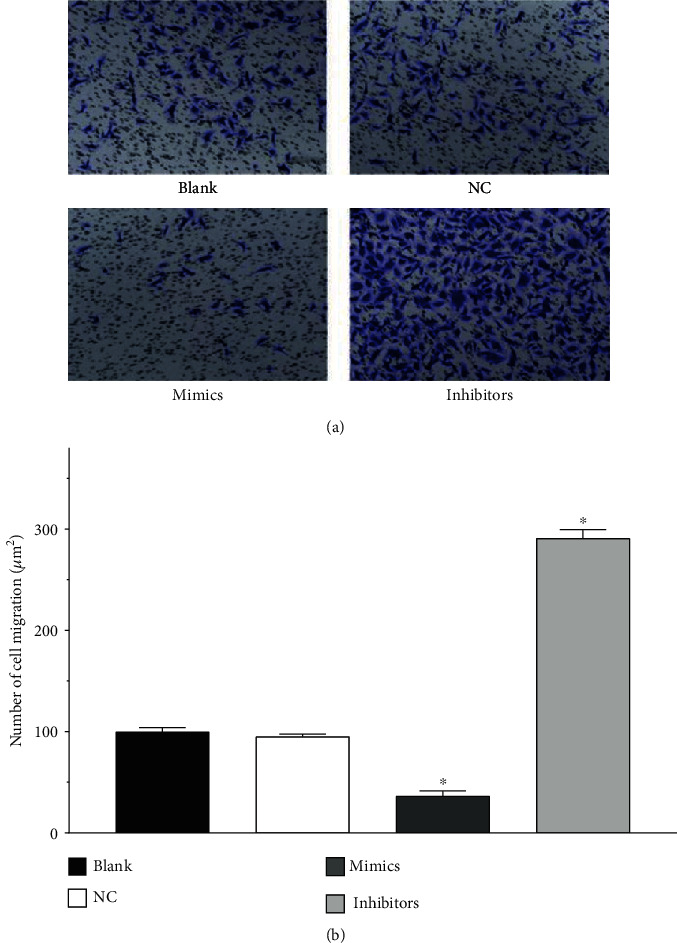
Effects of transfected miR-499a-3p on the migration of primary HUVECs revealed by Transwell tests. (a) Visual field (magnitude: 100x) of equal area was randomly chosen and schematically presented for each group, including blank, NC, mimics, and inhibitors groups. (b) The average number of cells counted in the visual field randomly chosen in each group (*n* = 3).

**Figure 6:**
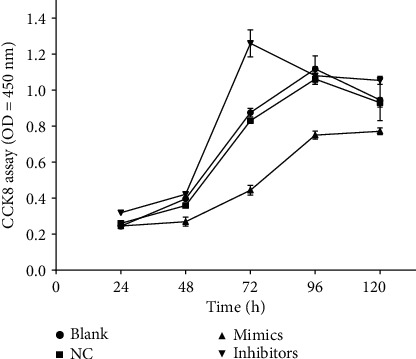
Effects of miR-499a-3p on the proliferation of primary HUVECs. The proliferation of HUVECs was measured by the CCK8 assays every 24 h after transfection of miR-499a-3p (*n* = 3). The OD_450_ is in proportion with living cell numbers and is representative of cell proliferation.

As shown in Table 1 and Figure 4. 24 h after scratching, no significant difference in the wound healing ability was observed between the blank and NC groups (*P* > 0.05). Compared to the NC group, the wound healing ability of the mimics group decreased significantly (*P* < 0.05) while that of the inhibitors group increased significantly (*P* < 0.05). Obviously, transfection with miR-499a-3p mimics weakened the wound healing ability of primary HUVECs while miR-499a-3p inhibitors enhanced it (see Table 1, Figures 5 and 6).

### 3.6. Transwell Migration Experiment Results

The average number of cells in the visual field of equal area randomly chosen was counted for each group, as shown in Figure 5. No significant difference was observed between the blank and NC groups (*P* > 0.05). Compared with the NC group, the average number of cells per visual field was significantly reduced in the mimics group (*P* < 0.05), whereas that of the inhibitors group was significantly increased (*P* < 0.05). These results suggested that miR-499a-3p can inhibit the migration of primary HUVECs, as shown in Figure 5.

### 3.7. Cell Proliferation

As shown in Table 2 and Figure 6, there was no significant difference between the blank and NC groups in OD_450_ values at each test time point (*P* > 0.05). In the time course of the test (24~72 h), the OD_450_ value of the miR-499a-3p mimics group is significantly lower than the NC group (*P* < 0.05) while that of the miR-499a-3p inhibitors group is significantly higher (*P* < 0.05). These results suggested that miR-499a-3p can inhibit the proliferation of primary HUVECs.

### 3.8. Flow Cytometry Assays

**Figure 7:**
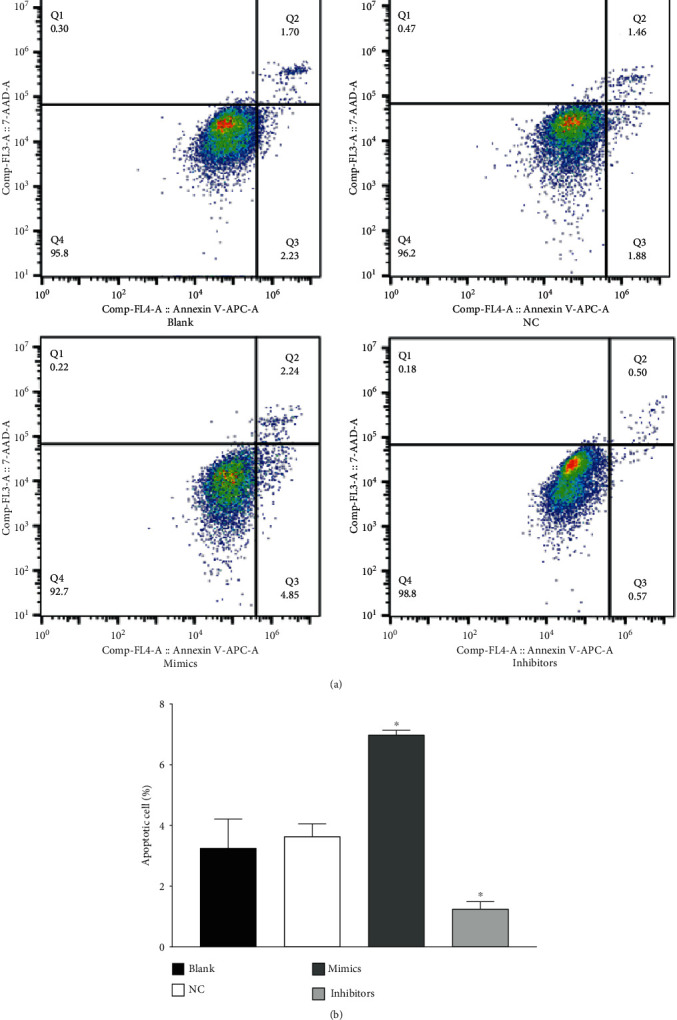
Apoptosis of primary HUVECs induced by transfected miR-499a-3p. (a) The effects of miR-499a-3p on the apoptosis of primary HUVEC determined by flow cytometry. Four groups were tested, including the blank, NC, mimics, and inhibitors groups. (b) Histogram show of the percentage of apoptotic cells in each group.

As shown in Figure 7, the percentages of apoptotic cells in the blank, NC, miR-499a-3p mimics, and inhibitors group are3.25 ± 0.91, 3.64 ± 0.42, 6.99 ± 0.15, and 1.25 ± 0.25 (*n* = 3), respectively. There was no significant difference between the NC group and the miR-NC group (*P* > 0.05). However, compared to the NC group, apoptosis in the miR-499a-3p mimics group significantly increased (*P* < 0.05) whereas that of the inhibitors group significantly reduced (*P* < 0.05). These results combined suggest that miR-499a-3p can modulate in the apoptosis of primary HUVECs and can promote the apoptosis of primary HUVECs.

## 4. Discussion

As a subtype of coronary atherosclerotic heart disease, unstable angina pectoris (UA) is well known for its high risk of death [24]. However, specific diagnostic markers for UA are still in absence. Considering the huge number of AMIs caused by UA per year, to find a new molecule marker, specific and sensitive, for clinical diagnosis and prognosis of UA is urgent.

In our previous work, we have found that the level of plasma miR-499a-3p within 2 h of chest pain is significantly increased in UA patients, compared to noncoronary heart disease patients with suspected angina pectoris (*P* < 0.05). miR-499 are the myocardial cell-specific precursors of miR-499-3p [25], located in the introns of the myosin gene *myh7b*, which is almost exclusively produced in the heart [26] and released into the blood from damaged myocardial cells during myocardial infarction [14, 27]. Thus, it is reasonable to suppose miR-499a-3p as a candidate marker for UA.

However, the past research studies on this question are in controversy. Zhang et al. reported that miR-499 appeared in the plasma as early as 1 h after the chest pain and continued to rise even 9 h later. And miR-499 is highly positively correlated with creatine kinase isoenzyme MB (CK-MB) and cardiac troponin I (cTnI), both of which can be detected only 2 h after chest pain in the AMI. The authors suggested that miR-499 can be used to diagnose AMI earlier and find out patients that would be missed in cTnI/CK-MB-aided diagnosis methods [28]. Shalaby et al. found that miR-499a appeared earlier in the circulation of UA patients than highly sensitive cardiac troponin T. And the Area Under Curve (AUC) of miR-499 for the diagnosis of UA patients was 0.98 (0.92-1, 95% CI, *P* < 0.001) which further supported the potential of miR-499 to be a new type of biomarkers for UA [29]. Chen et al. also reported a significant (*P* < 0.01) high level of miR-499 (5.12 ± 2.29) in the plasma of AMI patients, compared to that of UA patients (2.75 ± 1.39) and healthy controls (0.50 ± 0.35) [27]. On the contrary, Oerlemans et al. reported higher levels of miR-499 in UA other than non-ST-segment elevation myocardial infarction [30]. Widera et al. [18] also found no difference in miR-499 levels between UA and AMI patients. A larger case-control study is needed.

On the other hand, our earlier research, in which the level of circulating miR-499a-3p in UA patients was significantly higher than that in normal people with chest tightness symptoms, is consistent with the studies of Shalaby et al. and Chen et al. and supports miR-499 to be a biomarker for early diagnosis and identification of UA. Since miR-499a can be released into circulation with microvesicles [29], the increased level of miRNAs-499 in the UA state may be due to its passive release from ischemic myocardial tissue.

The pathogenesis of UA may shed light on the search for such a marker. Nowadays, UA is attributed to the rupture of atherosclerotic plaques and thrombosis, which are closely related to the dysfunction of vascular endothelial cells [31, 32]. It is clear that abnormal proliferation and migration in the vascular endothelium will impair the metabolic balance of vasoactive substance and promote thrombosis, all of which are vital to coronary vasospasm and acute myocardial ischemia. Furthermore, impaired vascular endothelial function will not only initiate the formation of atherosclerotic plaques but also play a central role in the production and progression of thrombosis, vasospasm, inflammation, and atherosclerosis [33]. Thus, markers which can weigh the function of the vascular endothelium would have great value for the diagnosis of UA.

In previous work, we have confirmed ADAM10 to be the target gene of miR-499a-3p by dual-luciferase assays [34]. ADAM10 is metalloproteinase located on the long arm of chromosome 15 and contains 17 exons. It is a type I transmembrane protein with multiple functional domains. It is not only involved in the hydrolysis of other membrane proteins but also participates in cell adhesion [9, 35], fusion, membrane protein shedding, and various cell signal transduction processes [36].

It has been confirmed that ADAM10 can affect the proliferation, migration, and apoptosis of endothelial cells to regulate AS. ADAM10 cuts vascular endothelial cadherin and acts on various substrates, including TNF-*α*, IL-6R, L-selectin, CX3CL1 [37, 38], IL-1*β*, NF-*κ*B, IL-8, Notch1, and epidermal cell growth factor [39–45]. Speck et al. found that fish oil can reduce the activity of ADAM10 in male LDLR-/- mice, which in turn counteracts the intimal lipoprotein infiltration and the accumulation of macrophages, which helps to improve the endothelial barrier function, indicating that downregulation of ADAM10 has anti-AS effects [46]. Yao et al. found that miR-25-3p derived from exosomes overexpressed by platelets can inhibit oxidized low-density lipoprotein- (ox-LDL-) induced coronary endothelial cell inflammation via inhibiting the NF-*κ*B signaling and ADAM10 expression [47].

In addition, ADAM10 can accelerate the apoptosis of normal smooth muscle cells and macrophages, thereby increasing the instability of atherosclerotic plaques [22, 23, 48]; it can also increase vascular permeability [49, 50], at the same time recruit leukocytes, accelerate leukocytes to cross the endothelial barrier into the arterial muscular layer, further promote the generation of macrophage-derived foam cells, and accelerate AS progress. Studies [51] have shown that ADAM0 can hydrolyze the receptors of the outer membrane of Notch protein, suggesting that in myocardial ischemia, ADAM10 may induce angiogenesis, inhibit cardiomyocyte apoptosis by activating the Notch signaling pathway, and play a role in improving myocardial ischemia. In addition, the study found that ADAM10 was significantly increased during the progression of AS plaques, and ADAM10 expression was relatively low in the vascular wall of healthy people and early human atherosclerotic lesions (literature), indicating that ADAM10 is involved in the formation of AS plaques.

However, there is little report on the relationship between ADAM10 and miR-499a-3p. Thus, in the present work, we tried to verify by experiments the possible relationship of miR-499a-3p with the function of vascular endothelium cells. As revealed by the function assessment after transfection of miR-499a-3p into primary HUVECs, upregulation of miR-499a-3p can inhibit the expression of ADAM10 and further inhibit the proliferation and migration of endothelial cells and promote the apoptosis of endothelial cells. Reverse results have been observed via downregulation of miR-499a-3p.

Our research is also consistent with other studies that miR-499a-3p is closely related to the proliferation and migration of vascular endothelial cells. Xu et al. compared the expression of miRNAs in serum samples from 13 atherosclerotic CAD patients and 5 healthy control subjects and found that circulating miR-499a-3p can inhibit the expression of MEF2C, thereby promoting the proliferation and migration of endothelial cells and smooth muscle cells [21]. Zhang et al. found that downregulation of miR-499 expression can increase PDCD4 expression by inhibiting the NF-*κ*B/TNF-*α* signaling pathway and protect endothelial cells from CAD inflammatory damage [52]. Liu et al. found that miR-499-3p activates the TLR4 signaling pathway by targeting IFNA2, inhibits the proliferation of diabetic retinopathy, and promotes retinal cell apoptosis. miR-499-3p may be a new therapeutic target for diabetic retinopathy [53].

Combined with the functions of ADAM10, this result revealed the effect of miR-499a-3p in the regulation of AS endothelial injury and further suggested the possible role of miR-499a-3p in the occurrence and development of AS. These results prefer miR-499a-3p to act as a molecular marker for early diagnosis of UA.

Early and accurate diagnosis of UA is key to reduce the risk of adverse events. Currently, common diagnostic indicators including creatine kinase isoenzyme and troponin are not sensitive and specific enough. It is necessary to find more sensitive and specific heart biomarkers. Circulating miRNAs could be used as potential biomarkers for CAD [19, 20, 54–57], since they are mainly released from ischemic necrosis or apoptotic cardiomyocytes [58]. Moreover, they can keep stable in different environments via binding to proteins to form complexes resistant to RNases [12, 15, 17, 59]. However, it should be carefully screened to make sure that the candidate miRNAs are specific and universal. Compared with traditional detection methods, miR-499a is released earlier in UA than troponin and CK-MB and lasts longer; and the levels of miR-499a in normal people, UA, and AMI patients have statistical differences [28]. Some studies have found that miR-499a combined with troponin and CK-MB will be more accurate in the diagnosis of acute coronary syndrome [29]. Therefore, we suggest miR-499a-3p could be used as a potential clinical marker for UA diagnosis.

In summary, miR-499a-3p may affect the occurrence and development of AS and participate in the pathological process of UA via inhibiting ADAM10 to regulate its downstream signaling pathways and cause endothelial cell dysfunction. It has the potential to be a new marker in early diagnosis of UA. However, more studies are needed to verify it, including comparing with the levels of troponin and CK-MB in a long period and studying the relationship between expression of ADAM10 and more functions of endothelial cells. All these experiments are still undergoing in our laboratory.

## Figures and Tables

**Table 1:** The migration ability of primary HUVECs affected by miR-499a-3p (*n* = 3).

Group	Scratch area (*μ*m^2^)		Healing area
	0 h	24 h	
Blank	520289.58 ± 16136.589	266445.83 ± 37869.965	253843.75 ± 29770.198
NC	503846.71 ± 19439.898	233287.45 ± 15020.513	270559.26 ± 34320.069^∗^
Mimics	387603.32 ± 20862.806	209321.5 ± 55904.856^∗∗^	178281.82 ± 45718.262^∗∗^
Inhibitors	607334.16 ± 26064.015	56818.06 ± 26411.427^∗∗∗^	550516.1 ± 26262.243^∗∗∗^

^∗^ *P* > 0.05, compared with the blank group; ^∗∗^*P* < 0.05, compared with the NC group; ^∗∗∗^*P* < 0.05, compared with the NC group.

**Table 2:** The OD_450_ values of different cell groups after transfection.

Group	24 h	48 h	72 h
Blank	0.244 ± 0.003	0.397 ± 0.013	0.875 ± 0.024
NC	0.260 ± 0.006	0.359 ± 0.055	0.831 ± 0.014
Mimics	0.245 ± 0.01	0.269 ± 0.025	0.444 ± 0.028^∗^
Inhibitors	0.319 ± 0.004	0.422 ± 0.005	1.26 ± 0.075^∗∗^

^∗^ and ^∗∗^, *P* < 0.05, compared with the NC group.

## Data Availability

The data used to support the findings of this study are included within the article. If there are any details needed, please contact the corresponding author.
